# The Blastocystis-colorectal cancer hypothesis: correlation is not causation

**DOI:** 10.12688/openreseurope.21693.2

**Published:** 2026-09-22

**Authors:** Özgür Kurt, Pauline D. Scanlan, Eleni Gentekaki, Lucy J. Robertson, David Carmena, Funda Dogruman-Al, Anastasios D. Tsaousis

**Affiliations:** 1 Acibadem University School of Medicine, Department of Medical Microbiology, Istanbul, Turkey; 2 APC Microbiome Ireland, University College Cork, Cork, Ireland; 3 School of Microbiology, University College Cork, Cork, Ireland; 4 University of Nicosia School of Veterinary Medicine, Nicosia, Cyprus; 5 Norwegian University of Life Sciences, As, Norway; 6 Spanish National Centre for Microbiology, Majadahonda, Spain, Spain; 7 Gazi University School of Medicine, Department of Medical Microbiology, Division of Medical Parasitology, Ankara, Turkey; 8 University of Kent School of Biosciences, Canterbury, England, UK; 9 Laboratory of Molecular and Evolutionary Parasitology, University of Kent School of Natural Sciences, Canterbury, England, UK

**Keywords:** Blastocystis, colorectal cancer, gut microbiome, correlation, causation

## Abstract

Although superficially persuasive, claims suggesting a causal link between *Blastocystis* and colorectal cancer (CRC) lack robust scientific support. As *Blastocystis* is the most common gut protist found in human populations globally, its detection in CRC patients is unsurprising and does not imply pathogenicity. Current claims championing a causal role for *Blastocystis* in CRC are based on speculative correlations, a single poorly controlled animal study, and inconsistent subtype associations. We argue that linking *Blastocystis* to CRC is premature, misleading, and may give rise to unnecessary concern in patients that are colonised by or test positive for *Blastocystis.* We emphasise the need for rigorously designed investigations to establish causal roles for any microorganism in disease and the importance of conclusions being based on solid evidence, particularly in matters of public health.

As active members of the European COST (Cooperation on Science and Technology) Action *Blastocystis under One Health* (CA21105) we wish to express our concerns regarding several statements presented in the review “An Update on *Blastocystis*: Possible Mechanisms of *Blastocystis*-Mediated Colorectal Cancer” by Tocci, Das, and Sayed. ^1^ Based on our collective expertise in *Blastocystis* research, we wish to note that no comprehensive epidemiological, *in vitro* or *in vivo* data conclusively supports the assertions of Tocci *et al.* ^1^ who suggest that a direct causal relationship between *Blastocystis* infection or colonisation and the development of colorectal cancer (CRC) has been established. Establishing causation requires carefully designed and integrated mechanistic and epidemiological studies with appropriate controls. Such studies currently do not exist, and the claims in the review of Tocci *et al.* ^1^ rely heavily on speculative interpretations of limited studies that we discuss in brief below.

*Blastocystis* is the most common protist in the human gut globally. More than 45 subtypes have been identified; over 10 of these are found in humans, with ST3 the most prevalent globally. ^2^ ^,^ ^3^ Despite the first description of *Blastocystis* being published more than 110 years ago, ^4^ its clinical significance is still under debate. ^5^ ^–^ ^7^ According to the available data, when *Blastocystis* is detected in clinical cases it is primarily associated with gastroenterological and dermatological conditions that are related to both the host’s immune status and other components of the gut microbiota. ^8^ ^–^ ^11^ Although some studies have reported an increased prevalence of *Blastocystis* among cancer patients, ^12^ ^,^ ^13^ these studies do not provide any evidence or mechanistic insights that would implicate a causal role for *Blastocystis* in CRC. For example, it could simply be that in CRC, alternations to the local environment, including the tumour microenvironment, which are associated with altered host physiology and immune function, as well as treatment-induced changes in gut ecology and broader dysbiosis, may create conditions that favour the colonisation and/or overgrowth of *Blastocystis.* This may explain an increased prevalence in cancer patients in some studies, however, future work should also include q-PCR assays to assess variation in *Blastocystis* abundance and potential overgrowth across different gut microenvironments and cohorts of interest. An association between *Blastocystis* and carcinogenesis has been shown in a rat model, where urinary oxidative indices correlated with carcinogenesis were shown to be higher in rats infected with *Blastocystis* than in non-infected rats. ^14^ However, to our knowledge, this is the only *in vivo* study reported in the literature and was conducted in laboratory rodents and not humans. The *Blastocystis* isolate used was ST3, the most common subtype occurring in humans, but not adapted to infect rodents, which typically harbour ST4. Most importantly, this isolate was obtained from a faecal sample of a non-symptomatic individual, concentrated on a density gradient and subsequently used to infect rats with the presence of other microbes (e.g. bacteria, viruses) in the concentrate not excluded.

Furthermore, the morphological forms presented in the study ^14^ appear to be inconsistent with recognised *Blastocystis* morphotypes. Given the marked pleomorphism of *Blastocystis,* ^15^ morphological variability may complicate reliable microscopic identification and classification, ^16^ potentially contributing to inconsistencies reported across studies rather than reflecting true biological differences. On this note, Tocci and colleagues ^1^ outline that certain *Blastocystis* subtypes are pathogenic. However, a detailed understanding of the genetic and phenotypic diversity of *Blastocystis* STs and their biological significance with respect to host health and disease is limited. Robust research data linking the disease-causing potential or pathogenicity of subtypes or morphotypes to CRC is simply not available. Based on the current literature, it is not possible to differentiate *Blastocystis* subtypes and/or strains as being either commensal or pathogenic, as contradictory study findings on *Blastocystis* subtypes and pathogenicity are widespread. ^15^ ^–^ ^18^

Contrary to studies suggesting a role for *Blastocystis* in CRC, a recent study using state-of-the-art metagenomic approaches that presented data from the largest cohort studied to date (involving more than 56,000 individuals from 32 countries) demonstrated a negative correlation between CRC and *Blastocystis.* ^19^ More generally, a negative correlation between *Blastocystis* and other diseases that involve inflammation of the gastrointestinal tract e.g. ulcerative colitis and Crohn’s Disease have been reported in independent studies. ^20^ ^,^ ^21^

## Conclusion

The history of microbial oncology, exemplified by *Helicobacter pylori* and gastric cancer ^22^ ^,^ ^23^ demonstrates that microbial involvement in cancer is possible. However, in those cases, causation was only established after decades of carefully controlled epidemiological, mechanistic, and clinical studies. For *Blastocystis*, such a body of evidence does not currently exist, and if we critically assess the evidence that is available at present, there is no credible basis for asserting that *Blastocystis* causes CRC in humans. The high prevalence of *Blastocystis* globally makes its detection in cancer patients unsurprising, and current studies provide, at best, speculative correlations. Importantly, premature claims of causation carry the risk of misleading clinicians, alarming patients, and diverting research away from more productive avenues. Furthermore, they undermine the nuanced view emerging from microbiome science, ^24^ ^–^ ^27^ where *Blastocystis* is often associated with gut health rather than disease. Future research may yet clarify the potential roles of *Blastocystis*, whether neutral, beneficial, or detrimental, but until such evidence exists, statements linking *Blastocystis* to cancer should be regarded as misleading and premature.

### Ethics and consent statement

Ethical approval and consent were not required.

## Disclaimer

The views expressed in this article are those of the author(s). Publication in Open.

Research does not imply endorsement by the COST ACTION funding body.

## Data availability statement

No data are associated with this article.
