# Supplementary material for: The Blastocystis-colorectal cancer hypothesis: correlation is not causation
Source: Open Res Eur. 2026 Sep 22;5:379. Originally published 2025 Dec 10. [Version 2] doi: 10.12688/openreseurope.21693.2 (PMC13032105; doi:10.12688/openreseurope.21693.2)
Supplement: Peer review history

# Reviewer report: Reviewer response for version 1

**DOI:** 10.21956/openreseurope.23467.r71489

**Published:** 2026-04-06

**Authors:** Bidisha Barat

**Affiliations:** 1 The University of Chicago, Chicago, Illinois, USA

**Major Concerns **

1. **Expand Discussion on Reverse Causality**
  While the manuscript suggests that CRC-associated changes may explain *Blastocystis* prevalence, this point could be strengthened by:
  - Elaborating on tumor-associated changes (e.g., hypoxia, immune modulation, mucus alterations)
  - Discussing how these changes may facilitate opportunistic colonization
2. **Clarify Morphological and Subtype Arguments**
  The critique of morphological inconsistencies is important but could be clearer:
  - Specify whether inconsistencies arise from methodological differences or misclassification
  - Clarify whether morphotypes are biologically meaningful or artifacts
  - Avoid overinterpretation given ongoing debates in the field
3. **Address Quantitative vs Qualitative Detection**
  The manuscript focuses on presence/absence data but would benefit from discussing:
  - The importance of *Blastocystis* load (e.g., qPCR-based quantification)
  - Distinguishing colonization from overgrowth
  - Potential threshold effects relevant to disease associations
4. **Integration with Broader Microbiome Context**
  While briefly mentioned, the manuscript could further integrate:
  - Interactions between *Blastocystis* and bacterial communities
  - Whether *Blastocystis* may act as a marker of a “healthy” microbiome rather than a driver

Where applicable, are recommendations and next steps explained clearly for others to follow? (Please consider whether others in the research community would be able to implement guidelines or recommendations and/or constructively engage in the debate)

Yes

Does the article adequately reference differing views and opinions?

Partly

Are all factual statements correct, and are statements and arguments made adequately supported by citations?

Yes

Is the rationale for the Open Letter provided in sufficient detail? (Please consider whether existing challenges in the field are outlined clearly and whether the purpose of the letter is explained)

Partly

Is the Open Letter written in accessible language? (Please consider whether all subject-specific terms, concepts and abbreviations are explained)

Yes

Reviewer Expertise:

Microbiome and colorectal cancer

I confirm that I have read this submission and believe that I have an appropriate level of expertise to confirm that it is of an acceptable scientific standard, however I have significant reservations, as outlined above.

# Reviewer report: Reviewer response for version 1

**DOI:** 10.21956/openreseurope.23467.r70107

**Published:** 2026-03-27

**Authors:** Sonia H Navia

**Affiliations:** 1 Universidad Nacional Autónoma de México, Tlalnepantla, Mexico

The letter addresses the controversial relationship between the gut protist *Blastocystis* and colorectal cancer (CRC). It argues that the current claim of a direct link between both conditions still lacks a solid scientific basis. The authors appropriately highlight the high persistence of *Blastocystis* in healthy populations, which makes its detection in CRC patients not entirely unexpected, but not necessarily evidence of a pathogenic role. In this sense, the manuscript offers a useful and timely counterpoint to premature causal interpretations.

 The authors clearly identify the main concern: the publication of a review (Tocci et al.) that suggests mechanisms of causality without definitive evidence. They also explain well why this can be problematic, as it may unnecessarily alarm colonized patients. Importantly, the manuscript does not ignore studies that support the pathogenicity hypothesis, but instead contrasts them with evidence from large meta-analyses and calls for more rigorous mechanistic and epidemiological studies.

 I do, however, have a few suggestions that could strengthen the manuscript:

- First, although CRC clearly changes the intestinal environment, the discussion could expand a bit more on how immunological, physiological, and morphological changes during cancer development may favor opportunistic colonization by *Blastocystis*. This would reinforce the idea that the association may be a consequence of the disease rather than its cause.
- Second, the authors criticize the inconsistent forms described in previous studies. This is an important point, but it would help to clarify whether this refers to problems in morphological identification, terminology, or interpretation across studies. Also, if the authors suggest that different forms may have distinct effects in the colon, that idea should be presented cautiously, since the biological relevance of several *Blastocystis* morphotypes is still debated.
- Finally, I would suggest that future studies should go beyond reporting simple PCR-based presence/absence and also include parasite burden, for example by qPCR. Given that the colon tumor microenvironment changes substantially, it is plausible that cancer may favor increased *Blastocystis* abundance without implying a causal role. In that sense, distinguishing colonization from possible overgrowth could help better frame the association and avoid overinterpretation.

Where applicable, are recommendations and next steps explained clearly for others to follow? (Please consider whether others in the research community would be able to implement guidelines or recommendations and/or constructively engage in the debate)

Yes

Does the article adequately reference differing views and opinions?

Yes

Are all factual statements correct, and are statements and arguments made adequately supported by citations?

Yes

Is the rationale for the Open Letter provided in sufficient detail? (Please consider whether existing challenges in the field are outlined clearly and whether the purpose of the letter is explained)

Yes

Is the Open Letter written in accessible language? (Please consider whether all subject-specific terms, concepts and abbreviations are explained)

Yes

Reviewer Expertise:

Cancer immunology, CRC microbiota

I confirm that I have read this submission and believe that I have an appropriate level of expertise to confirm that it is of an acceptable scientific standard.

# Response

**Published:** 2026-06-11

**Authors:** Pauline Scanlan

**Affiliations:** Microbiology and APC Microbiome Ireland, University College Cork Faculty of Science, Cork, County Cork, Ireland

**Reviewer comments:** The letter addresses the controversial relationship between the gut protist *Blastocystis* and colorectal cancer (CRC). It argues that the current claim of a direct link between both conditions still lacks a solid scientific basis. The authors appropriately highlight the high persistence of *Blastocystis* in healthy populations, which makes its detection in CRC patients not entirely unexpected, but not necessarily evidence of a pathogenic role. In this sense, the manuscript offers a useful and timely counterpoint to premature causal interpretations. The authors clearly identify the main concern: the publication of a review (Tocci et al.) that suggests mechanisms of causality without definitive evidence. They also explain well why this can be problematic, as it may unnecessarily alarm colonized patients. Importantly, the manuscript does not ignore studies that support the pathogenicity hypothesis but instead contrasts them with evidence from large meta-analyses and calls for more rigorous mechanistic and epidemiological studies.

Reply: Thank you for you very positive comments on our review and your constructive and important feedback.   I do, however, have a few suggestions that could strengthen the manuscript:

1. First, although CRC clearly changes the intestinal environment, the discussion could expand a bit more on how immunological, physiological, and morphological changes during cancer development may favor opportunistic colonization by *Blastocystis*. This would reinforce the idea that the association may be a consequence of the disease rather than its cause.

*Reply: This is an important point, and it is likely that changes in the disease intestine relative to a healthy one does favor the growth of certain microbial species. However, our understanding of specific abiotic and biotic factors that may support or inhibit Blastocystis growth are relatively limited in both health and disease contexts. As such we feel that perhaps the most prudent approach here is to emphasize this point a little more strongly perhaps but not go into specific details about what these factors might be as we simply do not have enough information. We have re written the following paragraph to emphasize this a little more and also to address your later point about qPCR and abundance, see paragraph below and main text lines 99-106:* *“It could simply be that in colorectal cancer (CRC) alterations to the local gut environment (including the tumor microenvironment) that are associated with altered host physiology and immune function, as well as treatment-induced disruption of gut ecology and broader dysbiosis may create conditions that favor the colonization and/or overgrowth of Blastocystis. This could explain the higher prevalence of Blastocystis observed in cancer patients in some studies and in addition to prevalence-based assays, future work should include q-PCR assays to assess Blastocystis abundance and potential overgrowth across different gut microenvironments and cohorts of interest.”*  

1. Second, the authors criticize the inconsistent forms described in previous studies. This is an important point, but it would help to clarify whether this refers to problems in morphological identification, terminology, or interpretation across studies. Also, if the authors suggest that different forms may have distinct effects in the colon, that idea should be presented cautiously, since the biological relevance of several *Blastocystis* morphotypes is still debated.

Reply: *Blastocystis* is a highly pleomorphic organism, as comprehensively described by Tan 2008 (PMID: 18854485), who outlines the main morphotypes (vacuolar, granular, amoeboid, and cyst), along with less frequently reported variants such as a vacuolar and Mult vacuolar forms. This marked morphological variability can make forms difficult to distinguish (particularly under routine light microscopy examination) and they may not always fit neatly into established categories. As highlighted by Stensvold 2015 (PMID: 25709945), there is also a lack of consensus regarding *Blastocystis* morphology, and some reported forms may represent artifacts. In practical terms, this variability complicates accurate microscopic identification and increases the risk of misclassification, especially in the absence of specialized expertise. Accordingly, when we noted that “the morphological forms presented in the study [14] appear to be inconsistent with recognized *Blastocystis* morphotypes,” our intention was not to question specific findings, but rather to suggest that discrepancies across studies may arise from methodological limitations in morphological identification. Therefore, we have revised the manuscript to reflect a more cautious interpretation, emphasizing that (i) *Blastocystis* exhibits substantial pleomorphism, (ii) this variability can hinder reliable microscopic detection and classification, and (iii) these challenges may contribute to inconsistencies reported across studies. This information has been now added in current lines 116-119 of the manuscript.  

1. Finally, I would suggest that future studies should go beyond reporting simple PCR-based presence/absence and also include parasite burden, for example by qPCR. Given that the colon tumor microenvironment changes substantially, it is plausible that cancer may favor increased *Blastocystis* abundance without implying a causal role. In that sense, distinguishing colonization from possible overgrowth could help better frame the association and avoid overinterpretation.

*Reply: We agree, and we have incorporated the importance of qPCR and quantitative data as outlined above, lines 99-106.*

# Reviewer report: Reviewer response for version 1

**DOI:** 10.21956/openreseurope.23467.r69716

**Published:** 2026-03-04

**Authors:** Camelia Munteanu

**Affiliations:** 1 University of Agricultural Sciences and Veterinary Medicine Cluj-Napoca, Cluj-Napoca, Romania

In this open letter, Kurt et al. emphasize that the implication that *Blastocystis* sp. may act as a causative factor in colorectal cancer (CRC) is not sufficiently supported by the current evidence. They present solid arguments outlining the limitations of the available studies and stress that further experimental and mechanistic research is necessary before any causal relationship can be established.

 Importantly, the authors caution against prematurely assigning a pathogenic role to *Blastocystis* sp. in CRC in the absence of robust supporting data. By carefully examining the existing literature, they challenge the assumption that this organism should be regarded as a confirmed etiological factor in colorectal carcinogenesis.

 Overall, this commentary is timely and relevant. It provides a balanced perspective on the topic and encourages a more cautious interpretation of the current findings. For these reasons, it is appropriate for Indexing.

Where applicable, are recommendations and next steps explained clearly for others to follow? (Please consider whether others in the research community would be able to implement guidelines or recommendations and/or constructively engage in the debate)

Yes

Does the article adequately reference differing views and opinions?

Yes

Are all factual statements correct, and are statements and arguments made adequately supported by citations?

Yes

Is the rationale for the Open Letter provided in sufficient detail? (Please consider whether existing challenges in the field are outlined clearly and whether the purpose of the letter is explained)

Yes

Is the Open Letter written in accessible language? (Please consider whether all subject-specific terms, concepts and abbreviations are explained)

Yes

Reviewer Expertise:

Cancer etiology, cancer biology, biomarkers, cancer omics analysis

I confirm that I have read this submission and believe that I have an appropriate level of expertise to confirm that it is of an acceptable scientific standard.
